# Do clinicians in areas of declining malaria transmission adhere to malaria diagnosis guidelines? A cross-sectional study from Kampala, Uganda

**DOI:** 10.1186/s12936-021-03729-8

**Published:** 2021-04-15

**Authors:** Angella Atukunda, Mwaka Amos Deogratius, Emmanuel Arinaitwe, Philip Orishaba, Moses R. Kamya, Joaniter I. Nankabirwa

**Affiliations:** 1 Department of Internal Medicine, School of Medicine, Makerere University College of Health Science, P.O Box 7072, Kampala, Uganda; 2 Infectious Disease Research Collaboration, Plot 2C Nakasero Hill Road, P.O Box 7475, Kampala, Uganda; 3 Clinical Epidemiology Unit, School of Medicine, Makerere University College of Health Science, P.O. Box 7072, Kampala, Uganda

**Keywords:** Malaria diagnosis, Declining transmission, Clinician practices

## Abstract

**Background:**

Uganda's clinical management guidelines recommend a malaria laboratory test in all patients presenting with fever (history of fever or an axillary temperature ≥ 37.5 °C), and only those with a positive test receive anti-malarial treatment. However, the current practice in areas with declining malaria transmission remains unknown. This study assessed the clinicians’ diagnostic practices, the factors associated with recommending a test, and the risk of missing a malaria case when a test is not recommended in patients presenting with fever in Kampala, an area of declining malaria transmission in Uganda.

**Methods:**

Between January and March 2020, 383 participants aged ≥ 12 years and presenting to Kisenyi Health Centre IV in Kampala district with fever were enrolled in the study. A questionnaire was administered during exit interviews, routine diagnostic practices were recorded from participant clinical notes, and a research blood slide was obtained for later reading.

**Results:**

Of the enrolled participants, 356 (93%) had a malaria diagnostic test recommended by the clinician. Factors associated with increasing prevalence of having a test recommended included; history of overnight travel (adjusted prevalence ratio [aPR] 1.07, 95% confidence interval [CI] 1.02–1.13, *p* = 0.011), being married (aPR = 1.07, 95% CI 1.01–1.13, *p* = 0.022), and having tertiary education (aPR = 1.09 95% CI 1.01–1.17, *p* = 0.031). Among the 27 participants where a malaria diagnostic test was not recommended, 4 (14.8%) had a positive study smear.

**Conclusion:**

Despite having significant declines in malaria transmission in Kampala in the last decade, clinicians at the study health facility highly adhered to the clinical management guidelines, recommending a malaria test in almost all patients presenting with fever. However, a significant proportion of malaria cases was missed when a test was not recommended. These results highlight the importance of laboratory testing for malaria in all patients who present with fevers and live in endemic settings even when the transmission has significantly declined.

## Background

A significant decline in the malaria burden has been realized in many sub-Saharan countries including Uganda in the last 10 years [1]. This has been attributed to the scale-up of effective malaria control interventions including; appropriate case management with artemisinin-based combined therapy (ACT), vector control using indoor residual spraying (IRS), and use of long-lasting insecticidal nets (LLIN) [1]. Although intervention coverage has expanded remarkably in Uganda over the past decade, progress on malaria control has been uneven in the country [2]. The 2018/2019 malaria indicator survey (MIS) shows a great variation in malaria burden between regions, with the lowest burden occurring in the urban and peri-urban areas of the central region and the highland areas of the southwestern region of the country [2–4]. The variability in the malaria burden in the country has been attributed to the geographical variation of the regions, different levels of urbanization, and differences in coverage of control interventions, especially IRS [2, 5, 6].

Malaria case management is one of the pillars of malaria control in Uganda and is guided by Uganda's national clinical guidelines. The current malaria treatment guidelines recommend that all patients presenting with fever (history of fever in the last 2 weeks or axillary temperature ≥ 37.5 °C) have a parasitological test for malaria with either microscopy or rapid diagnostic test (RDT) conducted, and anti-malarial treatment is only provided for those that test positive [1]. Since the rollout of these guidelines in 2015 [1], the country has observed progressive improvement in the uptake of the recommendations from as low as 17% in 2009 [6] to 51% in 2017/2018 [2]. Despite these improvements, several studies have shown that many patients with fever are still presumptively treated for malaria, while in other patients with fever, malaria is not considered as a differential diagnosis and no malaria diagnostic test nor malaria treatment is provided [7, 8]. Several factors have been associated with this observed non-adherence to diagnostic guidelines including; inadequate training, lack of supervision, absence of diagnostic services/ tests, and mistrust of the test results [7, 9–11].

A decline in malaria burden may be one of the reasons clinicians may not request a malaria diagnostic test in patients with fever, and although this may save on the costs of the diagnostics,

a malaria case may be missed at the first clinician-patient encounter, increasing the risk of progression to severe disease. This study assessed the clinicians’ malaria diagnostic practices, factors associated with recommending a malaria test, and the risk of missing a malaria case in patients not recommended a test among patients presenting with fever in a public health facility in Kampala, an urban district in Uganda that has had a significant decline malaria transmission in the last ten years.

## Methods

### Study setting

The study was conducted at the medical outpatient department of one public health facility in Kampala district (Kisenyi Health Centre IV). Malaria transmission in the Kampala district is very low, according to the 2018/2019 Malaria Indicator Survey, the parasite prevalence among children under 5 years is estimated at < 1% in the district [2]. Kisenyi Health Centre IV is the biggest and busiest public health centre in this district. It provides free health care and has a wide catchment area covering patients from the Kampala district, as well as patients from the surrounding districts of Wakiso, Mpigi, and Mukono. The medical Out-patient Department is managed by 12 clinicians including 3 medical doctors and 9 clinical officers and receives approximately 80 patients a day.

### Study design and participants’ enrolment

A cross-sectional survey was conducted at the out-patient clinic of Kisenyi Health Centre IV between January and March 2020. All patients presenting to the clinic were consecutively screened for eligibility to participate in the study. Study participants were enrolled if; (1) they were aged 12 years and above; (2) had a documented fever (history of fever the past 48 h or axillary temperature ≥ 37.5 °C); (3) they were able to speak English or Luganda; (4) provided written informed consent (parental consent and assent for patients aged 12–17 years). Exit interviews were conducted on all enrolled participants using a detailed questionnaire. The questionnaire captured information on the participant’s demographics, LLIN use the night before the survey, history of overnight travel, and malaria treatment history. Information on presenting complaints, routine diagnostic procedures, and laboratory results were recorded from the patient-held records and a research blood smear was collected by a finger prick from all the participants for malaria microscopy.

### Laboratory evaluations

All study thick blood smears were stained with 2% Giemsa for 30 min and evaluated for the presence of asexual parasites. Parasite densities were calculated by counting the number of asexual parasites per 200 leukocytes (or per 500, if the count was less than 10 parasites per 200 leukocytes), assuming a leukocyte count of 8,000/µl. A thick blood smear was considered negative if examination of 100 high power fields revealed no asexual parasites. For quality control, all slides were read by a second microscopist, and a third reviewer settled any discrepant readings.

### Statistical analysis

All data were collected using standardized case record forms and entered into Microsoft Excel. Analyses were performed using Stata, version 14 (Stata Corporation, College Station, Texas, USA). Baseline characteristics of the study population were summarized as percentages. The primary outcome was the prevalence of having a parasitological diagnosis recommended, and was defined as having a malaria blood slide or a malaria RDT recommended by the clinician divided by the total number of patients presenting with a fever or history of fever that were enrolled into the study. Individual factors associated with having a diagnostic test requested were assessed using modified Poisson regression with robust standard errors and expressed as prevalence ratios. At multivariate analysis, all independent variables with a p-value of less than 0.2 were entered into a multivariate model, and logical model building was used to eliminate variables. Both confounding and interaction were assessed during the model building. Prevalence ratios with their 95% confidence interval and p-values are presented and a p-value of < 0.05 was considered significant.

## Results

### Characteristics of the study participants

**Fig. 1:**
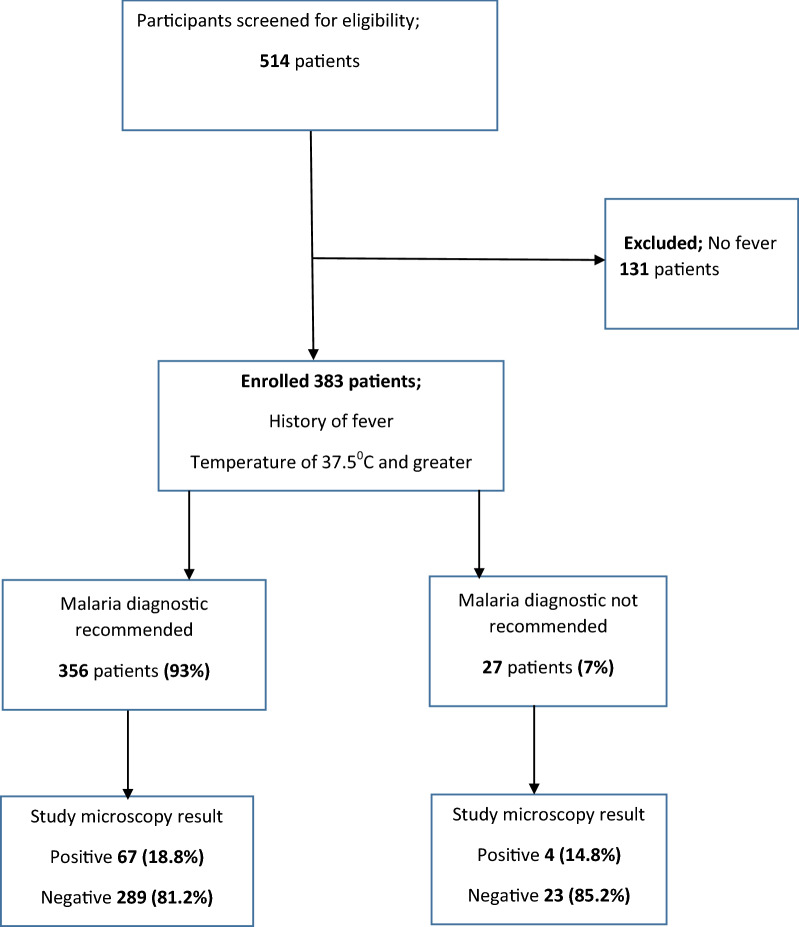
Study participant flow

Between January and March 2020, 514 patients were screened for eligibility to join the study, and 383 (74.5%) were enrolled. The only reason for exclusion for those screened was the absence of fever (131, 100%), as shown in Fig. 1. The median (interquartile range [IQR]) age at enrolment was 24 (18–31) years. Of the enrolled participants, 215 (56%) were female, 245 (64%) reported using a bed net a night before the survey, and 57 (15%) reported having used an antimalarial within the last 28 days before presenting to the facility. Out of the 383 participants, 173 (45.2%) reported a history of overnight travel within the last one month prior to presentation, and among those that travelled, 71 (41.0%) reported travel out of Kampala district. Details of the characteristics of the study participants are presented in Table 1.

**Table 1:** Descriptive characteristics of 383 patients presenting with fever at Kisenyi Health Centre IV

Characteristic	Frequency (N = 383)	Percentage (%)
Age categories		
< 18	83	21.7
≥ 18	300	78.3
Sex		
Male	168	43.9
Female	215	56.1
Division		
Central	59	15.4
Lubaga	104	27.2
Makindye	121	31.7
Kawempe	22	5.8
Nakawa	9	2.4
Out of Kampala	67	17.5
Marital status		
Single	258	67.4
Married	125	32.6
Education level		
Primary or lower	150	39.2
Secondary	193	50.4
Tertiary	40	10.4
Bed net use (the night before the survey)		
No	138	36.0
Yes	245	64.0
History of overnight travel		
No	210	54.8
Yes	173	45.2
Overnight travel details		
None Travel within Kampala district	210 102	59.0
Travel out of Kampala district	71	41.0
Last malaria infection or treatment		
Never	29	7.6
< 28 days	57	14.9
> 28 days	297	77.5

### Factors associated with recommending a malaria diagnostic test for a patient with fever

Of the 383 participants, 356 (93%) had a malaria diagnostic test recommended by the clinician. The results of the bivariate and multivariate analyses of the factors significantly associated with having a malaria diagnostic test recommended are shown in Table 2. Only variables that presented a significant association with having a malaria diagnostic test recommended in the multivariable analysis are presented below; being married, having a higher level of education, and having a history of overnight travel.

**Table 2:** Factors associated with a clinician recommending a malaria diagnostic test

Characteristic	Percent requested (n/N)	Unadjusted Prevalence Ratio (95%CI)	PP-value	Adjusted Prevalence Ratio (95%CI)	*P*-value
Age categories					
< 18	91.6 (76/83)	Reference			
≥ 18	93.3 (280/300)	1.02 (0.948–1.110)	0.603	0.97 (0.890–1.057)	0.486
Sex					
Male	95.2 (160/168)	Reference			
Female	91.2 (196/215)	0.96 (0.907–1.010)	0.110		
Marital status					
Single	92.0 (217/236)	Reference			
Married	96.8 (121/125)	1.06 (1.011–1.117)	0.017	1.07 (1.010–1.130)	0.022*
Education level					
Primary or lower	90.0 (135/150)	Reference			
Secondary	94.3 (182/193)	1.05 (0.983–1.117)	0.151	1.05 (0.989–1.124)	0.103
Tertiary	97.5 (39/40)	1.08 (1.007–1.165)	0.032	1.09 (1.007–1.172)	0.031*
Bed net use (the night before the survey)					
No	90.6 (125/138)	Reference			
Yes	94.3 (231/245)	1.04 (0.978–1.108)	0.206		
History of overnight travel					
No	90.0 (189/210)	Reference			
Yes	96.5 (167/173)	1.07 (1.017–1.131)	0.010	1.07 (1.016–1.130)	0.011*
Last malaria infection					
Never	89.7 (26/29)	Reference			
< 28 days	93.0 (53/57)	1.04 (0.899–1.196)	0.617	1.03 (0.881–1.197)	0.734
> 28 days	96.7 (89/92)	1.04 (0.916–1.182)	0.544	1.02 (0.884–1.179)	0.779

*Shows statistical significant results

Participants that were married were more likely to have a malaria test recommended than those that were not married (adjusted prevalence ratio [aPR] = 1.02, 95% CI 1.01–1.13, *p* = 0.022). In addition, participants with tertiary education were more likely to have a malaria diagnostic test recommended than those with primary/no education (aPR = 1.09, 95% CI 1.01–1.17, *p* = 0.031). Finally, participants who reported a history of overnight travel had a higher prevalence of a malaria diagnostic test recommended than those with no history of travel (90.0 vs. 96.5%, aPR = 1.07, 95% CI 1.02–1.13, *p* = 0.011). The participant’s age and treatment for malaria within the last month were not associated with recommending a diagnostic test during the visit in this study. Details of the factors associated with recommending a diagnostic test are presented in Table 2.

Of the 4 participants who tested positive on study microscopy and had not been offered a malaria diagnostic, half were teenagers and all were young people, half of them slept under mosquito nets and all of them had other diagnoses explaining their symptoms. Malaria test positivity rates among patients in whom malaria test was not recommended.

Of the 383 participants in the study, 27 (7.1%) did not have a malaria test recommended. The majority of the participants who were not recommended a malaria test were aged 18 years and above (20 [74.1%]), had no history of travel (21 [77.8%]), and had been treated for malaria within the last 28 days (20 [74.1%]). The study smear was positive in 4/27 (14.8%) of the participants in whom a clinical test was not recommended, and in 67/356 (18.8%) of the participants who had a smear recommended by the clinician (PR = 1.27 95% CI: 0.500–3.223, *P* = 0.607). Of the 4 participants, only one had received anti-malarial drugs in the last 28 days, 2 (50%) had reported using a bed net a night before the survey, and two reported a history of over-night travel in the last 60 days before the survey (Table 3).

**Table 3:** Characteristics of the 4 patients that were not recommended a test but tested positive on study microscopy

	Patient 1	Patient 2	Patient 3	Patient 4
Age (years)	12	13	22	35
Sex	Female	Male	Female	Female
Residence	Lubaga	Lubaga	Lubaga	Central
Reported net use	No	No	Yes	Yes
Marital status	Single (Child)	Single (Child)	Single	Married
Education level	Primary	Primary	Primary	Secondary
H/O Over-night travel	No	No	Yes	Yes
H/O anti-malarials use	No	Yes	No	No
Clinician’s diagnosis	URTI	Pneumonia	Bacteremia	UTI

*URTI* Upper respiratory tract infection, *UTI* Urinary tract infection, *H/O* History of

## Discussion

The study assessed the clinicians’ malaria diagnostic practices, factors associated with recommending a malaria test, and the risk of missing a malaria case when patients presenting with fever or history of fever in an area of declining malaria transmission are not recommended a malaria test. This is the first study that looked at clinicians’ malaria diagnostic practices and the risk of missing malaria in an area of declining malaria transmission. The findings indicate that a large proportion of patients with fever or a history of fever are still recommended a malaria diagnostic test despite declining transmission. The patient factors that were associated with a clinician recommending a malaria diagnostic test in this study included; being married, having a higher level of education, and having a history of recent overnight travel. Although few participants were not recommended a malaria diagnostic test by the clinician, the risk of missing a malaria case when a diagnostic test was not recommended was high. These findings suggest that clinicians in the Kampala district still highly adhere to the malaria clinical management guidelines despite the declining malaria burden, although in the few cases where a malaria diagnostic test is missed, chances of missing a malaria case are high.

### Clinician’s malaria diagnostic practices

The current malaria clinical guidelines were rolled out in 2016 when the burden of malaria was fairly high in Uganda [12]. However, despite the reductions in the burden especially in the urban centres including Kampala district [2], and the heavy workload at Kisenyi Health Centre IV, clinicians at this study clinic highly adhered to the national malaria clinical management guidelines. The guidelines recommend that all patients with fever should receive a malaria diagnostic test before treatment is initiated [1]. In this study, nine out of every ten participants with fever were recommended a malaria diagnostic test. Having a malaria test done in patients living in a malaria-endemic country and reporting health facilities with fever is important as it; (1) reduces the risk of overdiagnosis of malaria when patients are presumptively treated, and thus reduces the risk of exposing patients to unnecessary side effects, treatment costs, and development of resistance to the available anti-malarials [13]; (2) it ensures that clinicians get the opportunity to investigate alternative causes of the patient’s disease, which results in improved clinical outcomes when patients are correctly managed [14, 15].

The high testing rates observed in this study are comparable to data from the World Health Organization (WHO) which shows that the percentage of patients with suspected malaria that was tested with either an RDT or microscopy was over 85% in 2018 in moderate and high transmission areas [16, 17]. However, the proportion in this study is higher than the recent national estimates for Uganda which were reported to be 64% in 2018 [17]. This finding is also higher than what was found in several other studies in Uganda where the testing rate is estimated to be between 51% and 65.5% [2, 7, 17]. The higher proportion observed in our study could be due to the regular Continuous Medical Education (CME) training about malaria diagnosis and management given to clinicians practicing at this facility, including one that was offered a few weeks prior to the study on-set. Indeed training has been shown to improve malaria case management in Uganda and elsewhere [18–20] and should be regularly done to improve patient care not only for malaria but for all diseases.

### Patient factors associated with recommending a test among patients with fever

The patient factors significantly associated with a managing clinician recommending a malaria diagnostic test included the participants’ marital status, history of overnight travel, and education level. Clinicians were more likely to recommend a malaria test in participants that reported a history of overnight travel than those who had no history of overnight travel. This could be because overnight travel is a well-recognized factor that increased the risk of malaria infections [21]. This increased risk has been linked to the changes in behaviour when people are away from home including; engaging in outdoor activities like communal drinking until late in the night and reduced use of malaria preventive measures like sleeping under a bed nets [22–24]. It is, therefore, not surprising that clinicians in this study were more likely to suspect malaria and request for a confirmatory test in fever patients that report over-night travel.

Second, clinicians were more likely to recommend a test if a patient had a higher level of education than when the education level was low. The positive association between health and education has been well documented [25–27], and although it has been mostly linked to self-reported good health, the theoretical explanation of work and economic conditions can be extended to the findings. Education is an important aspect of the socioeconomic status and well-educated people have resources to allow them to seek the right care, challenge the care if not satisfactory, and pay for care (despite the fact that the diagnostic tests were free of charge at this facility) if recommended and not free of charge. Besides the social status, educated people have a high sense of personal control and are more likely to be aware of the minimum standards of care as recommended by the national guidelines. With this in the background, it makes sense that when clinicians interface with an educated person, they are more likely to follow standard guidelines as observed in this study.

Finally, married participants were more likely to have a test recommended by the managing clinicians compared to participants who were not married. This can also be explained by the increase in the social support that people who are married have over the single participants in this setting. With this support, married people have better structures that enable them to be informed and supported to seek the right care. With this empowerment, clinicians will likely adhere to guidelines when taking care of them than when the support structures are absent. This finding is similar to what was observed in a study in Tanzania where a high social-economic status was associated with better malaria-related outcomes [28].

### Risk of missing malaria cases when a test is not recommended

Despite the low burden of malaria in the study area, the risk of missing a malaria case was high when patients with fever were not recommended a malaria test. These results show that even when the transmission has significantly declined, it is important to rule out malaria when patients living in endemic countries present with fever. Failure to diagnose malaria in febrile patients puts them at risk of progression to severe malaria, and other potentially fatal malaria complications [29]. In addition, these patients end up receiving ineffective treatment of the febrile episode, resulting in prolonged ill health that necessitates repeated visits to health facilities. This not only generates an economic but also a social burden that disproportionately affects the individual and their families [30, 31].

This study was not without limitations. First, the study was conducted during the high-transmission season following a Christmas break and during the rains, and might not represent case management patterns during other times of the year. Traditionally, a spike in malaria cases is observed following the festive seasons and this could be the reason the testing rates observed in this setting were higher than what has been observed in other studies. Secondly, the study was carried out at one centre and the results may not represent what is happening at other facilities in Kampala and thus may limit generalizability. However, there is confidence that given that Kisenyi Health Centre IV is the largest and busiest health centre in the district, the results provide a fair representation of the care in facilities with similar settings in the Kampala district. Third, study results might have been subject to bias from the “Hawthorne” effect, whereby health workers perform better when they are aware their actions are being studied.

## Conclusion

Despite having significant declines in malaria transmission in Kampala in the last decade, clinicians at the study health facility highly adhered to the clinical management guidelines, recommending a malaria test in almost all patients presenting with fever. However, a significant proportion of malaria cases was missed when a test was not recommended. These results highlight the importance of laboratory testing for malaria in all patients who present with fevers and live in endemic settings even when the transmission has significantly declined. More still, a malaria test should always be recommended even when other diagnoses like UTI, pneumonia or bacteraemia are suspected.

## Data Availability

The datasets used and/or analysed during the current study are available from the corresponding author on reasonable request.

## Abbreviations

ACT: Artemisinin-based combined therapy
aPR: Adjusted prevalence ratio
CI: Confidence interval
CME: Continuous Medical Education
IRS: Indoor residual spraying
KCCA: Kampala City Council Authority
LLIN: Long-lasting insecticidal net
MIS: Malaria indicator survey
RDT: Rapid diagnostic test
SOMREC: School of Medicine Research and Ethics Committee
